# Experiences of Sexual Harassment by Patients among Nurses at the Mental Hospital of West Java Province: A Qualitative Study

**DOI:** 10.3390/ijerph20085525

**Published:** 2023-04-14

**Authors:** Iyus Yosep, Rohman Hikmat, Suryani Suryani, Ai Mardhiyah

**Affiliations:** 1 Department of Mental Health, Faculty of Nursing, Universitas Padjadjaran, Bandung 40132, Indonesia; 2 Faculty of Nursing, Universitas Padjadjaran, Bandung 40132, Indonesia; 3 Department of Pediatric Nursing, Faculty of Nursing, Universitas Padjadjaran, Bandung 40132, Indonesia

**Keywords:** nurses, sexual harassment, workplace

## Abstract

Sexual harassment is behavior related to forced sex without the consent of the victim. Incidents of sexual harassment on nurses can be in the form of physical behavior and verbal behavior. The factor of power relations between men and women and the patriarchal culture in Indonesia are the causes of the sexual harassment of mental health nurses, so many incidents of the sexual harassment of women occur. The forms of sexual harassment that occur include kissing, hugging from behind, and verbal abuse related to sex. The purpose of this study was to explore the experience of sexual harassment of psychiatric nurses at the Mental Hospital of West Java Province. This study implemented a qualitative descriptive approach using the NVIVO 12 software application. The sample in this study was 40 psychiatric nurses at the Mental Hospital of West Java Province. The sampling technique in this study was focus group discussions with semi-structured and in-depth interviews. The data analysis in this study used a thematic analysis. This study shows that sexual harassment behavior is carried out by patients in physical and verbal forms. Sexual harassment is mostly carried out by male patients towards female nurses. Meanwhile, sexual harassment took the form of hugging from behind, kissing, naked patients in front of nurses, and disturbing nurses with verbal abuse related to sex. Nurses feel disturbed, afraid, anxious, and shocked by the incidents of sexual harassment committed by patients. Sexual harassment by patients towards nurses causes psychological problems for nurses and causes nurses to want to leave their jobs. Gender adjustment between nurses and patients is a preventive effort to prevent the sexual harassment of nurses. Sexual harassment by patients causes a decrease in the quality of work of nurses in providing nursing care, creating a work environment that is less safe and comfortable.

## 1. Introduction

Sexual harassment is an act related to unwanted, impolite, and unpleasant sexual matters [1]. Sexual harassment is also defined as behavior related to sex that is considered by the victim to be something that offends, exceeds their power, and interferes with their well-being [2]. Some of the factors that can cause sexual harassment to occur in the workplace are personal characteristics and job characteristics. Individual characteristics such as age (young), personality, and appearance can encourage someone to take actions that are unpleasant to that individual [3]. Forms of sexual harassment include whistling, flirting, remarks with sexual overtones, showing pornographic materials and sexual desires, poking or touching body parts, and gestures of a sexual nature resulting in discomfort, offense, and feeling degraded and possibly causing health and safety issues [4]. Perpetrators of sexual harassment can commit sexual harassment anywhere, including in the workplace.

Incidents of violence against nurses have increased in the workplace. Nurses are three times more at risk than other occupational groups to experience workplace violence [5]. According to a report by the International Labor Organization (ILO), the incidence of sexual harassment among nurses is higher than that of other health workers. Nurses in Indonesia provide nursing care for 24 h (three shifts), so there is a risk of sexual harassment because they interact a lot with patients [6]. According to the World Health Organization (WHO), sexual harassment of psychiatric nurses occurs because of the aggressive behavior of mental patients, which causes sexual problems [7]. Incidents of sexual harassment against nurses are often found in the aggressive behavior of patients and their staff [8].

The prevalence of sexual harassment has increased in the workplace. Previous studies in Asia, Europe, and the Middle East reported that as many as 17.9% of nurses experienced sexual harassment at work [9]. Incidents of sexual harassment are more common in developing countries in the Asian region. Previous studies also showed that there was an increase in the incidence of sexual harassment in 2018 of as much as 5% from 2017 [7]. The sexual harassment that often occurs to nurses includes kissing and patients suddenly hugging from behind without the nurse’s consent.

As a result of acts of sexual harassment towards nurses, nurses feel sad (86%) and disappointed (79.3%), leave their jobs as nurses for fear of their work safety (18%), consider resigning from their jobs (15%), and deal with acute stress, post-traumatic stress symptoms, decreased work productivity, physical injury, and death [10]. Sexual violence has a number of physical and psychological effects, including physical injury, migraine and tension headaches, guilt, and the loss of self-confidence and belief in one’s abilities, as well as detrimental effects on the quality of patient care. Other impacts of sexual violence are related to work, such as lost working days, limited activities, the termination of employment, changing jobs, and even the quality of patient care [11]. Therefore, it is very important that every individual is informed in advance how to react in the event of sexual violence.

More than half of nurses in Taiwan reported having experienced physical violence and verbal abuse [9]. A previous study found that 25% of nurses experienced sexual harassment in the workplace [12]. Rates of exposure vary by world region (Asia, Europe, and the Middle East), with the highest rates for physical violence and sexual harassment in the Asian region, and the highest rates of non-physical violence and bullying are found in the Middle East. In addition, other data show that as many as 25% of psychiatric nurses in Indonesia have experienced trauma due to sexual harassment in mental hospitals [12].

Previous research that reported the sexual harassment of nurses was carried out on 277 nurses. In this study, 8% of nurses experienced sexual harassment, which, on average, was perpetrated by men (71%) in the form of verbal violence in the form of sexist comments about clothing, bodies, and sexual activity [12]. Another study found that 90% of nurses experienced one type of sexual harassment and 33% of respondents experienced severe sexual harassment. A study of 542 nurses showed that around 52% of nurses had experienced sexual harassment of various types, including verbal, physical, and visual harassment [13].

Sexual harassment has a major negative impact on nurses. The physical impact is in the form of damage to the physical condition of the nurse, while the psychological impact is in the form of anxiety, depression, low self-esteem, and social isolation [9]. Sexual harassment can also put nurses at risk of suicide [14]. Incidents of high-risk sexual harassment occur in mental hospitals that require intensive care. Therefore, the authors were interested in exploring the experiences of nurses who experience sexual harassment in mental hospitals using a qualitative study design.

## 2. Materials and Methods

### 2.1. Study Design

This study used a descriptive approach with a qualitative study design [15]. This design aimed to explore various situations and circumstances experienced by the respondents, including those that had been and were being experienced by the respondents [16]. This study aimed to explore the experience of sexual harassment of psychiatric nurses at the Mental Hospital of West Java Province. 

### 2.2. Research Characteristics and Reflexivity

This study was conducted using semi-structured interviews through focus group discussions (FGD) [15]. At the beginning, the authors explained the title, purposes, and benefits of the study to the respondents; that the study was designed to provide them with an understanding of the purposes and benefits; and that they would not be harmed by the study to be conducted. Informed consent was explained to the respondents. The respondents had the right and freedom to withdraw from the research. The respondents could also answer or not answer questions that made them uncomfortable. The authors also maintained the privacy of the respondents during the interviews. All recorded data were anonymized using coding. The respondents in this study were volunteers, and this study did not allow the respondents to experience physical or psychological harm. The authors were obliged to respect the privacy of the respondents by keeping their information confidential. Consequently, the researchers did not include the names of the respondents on the data collection sheet, except their initials. Interviews were conducted by researchers who had experience in the field of psychiatric nursing and had published articles on psychiatric nursing.

### 2.3. Participants and Setting

This study used non-probability purposive sampling to select the participants. Purposive sampling includes different types of sampling techniques, from homogeneous sampling to critical case sampling [17], that can be used to achieve a qualitative research design [18].

The inclusion criteria for this study were mental health nurses with a minimum educational background of diploma III, the lowest education level to become a nurse in Indonesia, that had worked for at least one year (nurses have a probation period for one year; after passing the probationary period, they are placed in the room) and had a past experience of exposure to sexual harassment. After selecting based on the characteristics of the participants and recommendations from the head of the nursing department, there were 40 nurses who responded to this study. For the selected participants, the first stage of a preliminary study consisted of a short interview with the nurses about the main problems occurring in the wards of mental hospitals in West Java, Indonesia. In the second stage, the respondents filled out closed questionnaires for screening and to determine if there had been traumatic experiences or exposure to sexual harassment. The third stage was the selection, by the ethics committee, of nurses who had experiences of sexual harassment and discussions with the head of the nursing department based on the inclusion criteria. Based on the results of the selected participants, 40 nurses were selected. This study was conducted on 24–25 August 2022 in Villa Istana Bunga Lembang, Bandung, Indonesia.

### 2.4. Ethical Considerations

The authors submitted a research proposal to the ethics committee of the Faculty of Medicine and Health Sciences, Universiti Malaysia Sarawak (UNIMAS), Malaysia. Then, after going through the review process from the ethical commission, this study received ethical approval from the Ethics and Research Commission of the Faculty of Medicine and Health Sciences, Universiti Malaysia Sarawak (UNIMAS), Malaysia. The ethics clearance number was UNIMAS/NC-21.02/03-02 Jld.2. Before conducting this study and collecting data, the authors submitted a research proposal to the Ethics Commission at the Mental Health Hospital of West Java Province. Approval of research implementation was given by the ethics committee within one week. Then, the researcher submitted a request for data collection to the education and training office. After obtaining permission from the education and training office, the researcher immediately completed the research administration to carry out data collection.

### 2.5. Data Collection

Focus group discussions (FGD) were carried out using semi-structured interviews to explore the experiences of sexual harassment of mental health nurses. The FGDs were conducted in groups (10 nurses/group), with the moderator being a lecturer in the Department of Mental Health, Faculty of Nursing, Universitas Padjadjaran. Data were recorded using an audiovisual camera. Focus group discussions were conducted over three sessions (60–90 min/session). The collected data were typed verbatim. The authors had made guidelines for asking for the qualitative data needed in this study. The following questions were asked during the FGDs: (a) Have you ever been sexually harassed by other health workers, patients, or family? (b) Give an example of the sexual harassment you have felt. (c) What sexual harassment behavior happens most often to you at the mental hospital? (d) What feelings or impacts arise when you experience sexual harassment violence? (e) What do you usually do after the occurrence of sexual harassment?

### 2.6. Data Analysis

The data analysis used a thematic approach. The software used to organize and categorize the data was the NVIVO 12 software application (QSR International) [19]. The stages of the analysis were as follows:

1. We listened to the FGD recordings, then made a transcript to understand the data as a whole;
2. We included non-verbal notes from the research respondents to gain a comprehensive understanding;
3. We read the transcript repeatedly and reflected on the contents of the transcript to ensure that the contents of the transcript matched the recording;
4. We identified the contents of the obtained data;
5. We grouped data based on the number of informants and explained the statements that emerged from the transcripts;
6. We reflected on the data obtained from the entire interview;
7. We wrote down the themes that emerged from the contents of the entire transcript and illustrated them according to the respondents’ statements;
8. We validated the obtained data by clarifying them with the informants.

We synthesized the existing statements so they did not conflict with the contents of the entire transcript.

## 3. Results

The demographic characteristics in this study are reported based on gender, educational level, duration of work, and the longest workplace. The respondents in this study were 40 psychiatric nurses at the Mental Hospital of West Java Province, Indonesia (Table 1).

The majority of the respondents were female (75%). The respondents were predominantly diploma III or nursing academy graduates (55%), and few of them were master’s graduates. The smallest number of the respondents held master’s degrees with psychiatric nursing specialties, i.e., only 10% of the 40 respondents. Meanwhile, the majority of the nurses (40%) had been working for 11–15 years. Only a small number (12.5%) of the mental health nurses had experience of more than 20 years. Of their entire working experience, the respondents were generally on duty in chronic rooms (35%).

### 3.1. Sexual Harassment Experiences among Nurses

The results of the content analysis show that sexual harassment by patients occurs towards nurses at the mental health hospital. The patients commit sudden sexual harassment such as hugging from behind, being naked in front of a nurse, forcibly kissing a nurse, seducing a nurse for sexual intercourse, and holding a nurse’s breasts. The incidents of sexual harassment mostly involve male patients harassing female nurses. Participants described their experiences of sexual harassment as follows:

*A-1—“I have read the medical records of patients who have a history of sexual abuse, so I keep my distance from these patients. But when I met the patient, he suddenly kissed my shoulder and asked me to have sex. I immediately left the patient because I was scared and shocked.”*

*A-6—“when I walked into the patient’s room, suddenly the patient hugged me. I was shocked, I was also surprised because the patient was naked. After that, I did not want to pass through the patient’s room”*

*A-8—"Unexpectedly, the patient approached me and touched my right breast. His hands groped my breasts.”*

*B-2—“Bipolar patients have insulted me for using red lipstick. When a patient is in the maniac-episode, he would yelled; let me kiss you, I want to kiss you. That experience made me not wanting to go to work.”*

*C-6—“When I entered the ward, my buttocks were suddenly held, touched, and groped from behind by a patient and I would immediately slapped him.”*

### 3.2. Nurses’ Experiences of Sexual Violence

The forms of sexual harassment towards psychiatric nurses were as follows:

1. Touching lightly with the fingertips;
2. Asking for sexual intercourse;
3. Accusing of being a whore and forcibly embracing;
4. Holding breast and buttocks;
5. Forcibly kissing while naked;
6. Compelling to get married and holding onto privates.

### 3.3. Nurses’ Responses to the Exposure of Violence

#### 3.3.1. Unpredictable

The word “suddenly” dominated the traumatic events encountered by nurses in mental hospitals. The repetition of the words “suddenly, startled, reflex, do not know” showed that the situation was unpredictable. For instance, when a patient’s hallucination suddenly emerged, he or she hugged, kissed, hit, threw, teased someone about having sex, and forced someone to do something involving sex. For more details, observe the descriptions of the data below:

*A-4—“When he was reminded to take medication, he suddenly kissed me; He suddenly pointed his hand to my veil and eyes.”*

*A-7—“A glass was suddenly thrown, hit my leg and immediately swelled up, then the patient tempted me to have sexual intercourse. “He bathed alone. I don’t know why he was spitting water from his mouth at me.”*

*A-8—“He is grumpy and very close to me, suddenly he hold breast and buttocks”.*

*B-4—“Suddenly he opened the door, I was hugged tightly by force, then I hit the patient, I didn’t realize what I had done.”*

*C-1—“Suddenly he attacked me, he grabbed my face, it was spontaneous and difficult for me to escape, but this veil and my shirt buttons got loose.”*

*D-1—“I was shocked when I gave medicine to patients. male patient asked me to have sex.”*

#### 3.3.2. Anticipatory Responses

In this study, it was found that nurses anticipate incidents of sexual harassment by patients. Nurses are careful, stay away from patients who have committed sexual harassment, and strengthen themselves. Detailed explanations of nurses’ anticipation are provided below:

*D-1—“I tend to be more emotional in an acute room, we have to in a predominant position firstly, to show who the boss at the room is, something likes at a prison.”*

*D-2—“We should be sure that we must firstly be strong. If the patients perceive that we are weak, we will forever be tortured and humiliated. We entered the room with some uncertainties…”*

*D-6—“For example, as an anticipatory action, I must be dare to interact with the patients”.*

*B-1—“While Mr. Jimi (my partner) cannot yell anymore, because he is below the patient’s position, at the time of trauma, we were scared of the patients, so anytime we met a patient we came to be fearful and cautious.”*

*C-8—“If I do not know the patient or there was a history that the patient has ever committed sexual violence behaviours I would kept a distance from the patient for safety reason.”*

*C-1—“I must be very careful. There were patients who try to suicide. I was very afraid of something like that again.”*

*D-4—“We must work carefully.”*

*D-3—“Before making a contact with patients, I have to briefly look for an exit way to escape.”*

*A-5—“When he hallucinate, he would strike me; it traumatized me and from now on I will not want to work in a psychiatric hospital anymore; I do not want to experience that trauma again.”*

*C-10 “I am still denying (refusing) that I’m working for a mental hospital.”*

  *“Every day I was scared of female patients. I felt uncomfortable. Frankly, if the patients met me, I’m still afraid until now.”*

## 4. Discussion

The results of this study indicate that nurses feel shocked by the incidence of sexual harassment by patients. Most of the incidents of sexual harassment were committed by male patients. Nurses need the ability to their control emotions when responding to sexual harassment by patients. In addition, nurses also receive sexual harassment through actions such as kissing, touching, and hugging from behind.

The sexual harassment of nurses is mostly carried out by male patients, and the nurses who become victims are female nurses. Therefore, male nurses are needed more than female nurses in mental hospitals for the prevention of violence against nurses. This is in line with previous studies that showed that more male nurses are needed in mental hospitals [12,20]. This aims to prevent the violence that occurs towards nurses. Previous studies have shown that incidents of violence against nurses are more common in female nurses [21,22]. The high rate of violence occurs because of power relations between men and women. Therefore, male patients feel more empowered to commit violence against women.

Incidents of sexual harassment against nurses often take the form of physical and verbal violence. Physical violence takes the form of hugging from behind and kissing. This is in line with previous studies that showed that 5% of incidents of sexual harassment against nurses were in the form of receiving hugs from patients and patients forcibly kissing nurses [13,23]. In addition, patients also sexually harass nurses in the form of physical insults and make sexual advances. Another study showed that female nurses were subjected to humiliation for their physical appearance and breasts by patients [24]. This causes physical and psychological problems due to sexual violence by patients.

Forms of sexual harassment against nurses can be in the form of physical and verbal abuse. Previous studies stated that physical abuse includes unwanted touching leading to sexual acts such as kissing, patting, hugging, pinching, stroking, massaging the nape of the neck, pressing against the body, and other physical touches [9,25]. Verbal harassment includes verbal remarks/unwelcome comments about one’s private life, body parts, or appearance, including jokes and sexually charged comments full of lust, gestures with fingers, licking lips, and other forms of sexual harassment, which are also visual forms that are often carried out by other workers. Visual harassment includes showing pornographic material in the form of photos, posters, cartoon images, screensavers, and others and harassment via email, SMS, and other modes of electronic communication. Psychological/emotional harassment includes persistent and unwanted requests and invitations, unwanted invitations to date, and insults of a sexual nature [11,26].

The psychological problems of nurses who experience sexual violence are fear and anxiety. Victims of sexual violence can also experience various interpersonal problems, such as distrust of others, difficulties in relationships, isolating themselves, and fear of men [27,28]. In addition, victims who experience severe psychological trauma are likely to have a strong urge to commit suicide [26,29]. This is supported by the results of a previous study that found that 1 in 5 victims of sexual violence had attempted suicide [30]. This number is greater than the number of suicide attempts made by victims of other crimes.

Previous studies have shown that almost 10% of nurses in Thai Hospitals experience sexual harassment [31]. Sexual harassment is mostly carried out by patients with mental disorders (50%) [32]. A patient committed sexual harassment because he had a history of drinking alcohol, a history of sexual violence, and a history of previous abuse [33]. This is in line with a previous study that showed that patients who have committed sexual harassment are at risk of becoming perpetrators of sexual harassment in the hospital [28].

The psychological impact can be considered a type of post-event trauma where this trauma is enough to affect the victim, especially causing excessive fear and anxiety as a result of the brain accidentally having flashbacks of violent incidents that have been experienced. Some people who have experienced trauma will feel anxious and very scared when they experience an incident that is similar to the violence they have experienced [34]. This cannot be avoided because this is one of the psychological effects of sexual violence.

Even though it has many negative impacts, not all women continue to perceive sexual violence as a negative experience. Some women can endure this crisis, even experiencing a more positive life afterwards [35]. The result of a previous study showed that victims of sexual violence experience several positive changes after sexual violence, such as being closer to family, feeling stronger and more optimistic, appreciating and being grateful for what they have, having more empathy for victims of sexual violence and others in general, and so on [13]. Another study found that most victims of sexual violence reported positive changes after the incident of sexual violence, such as becoming stronger, more careful, closer to their mothers, and more empathetic towards victims of sexual violence and raising children with a higher awareness of sexual responsibility [36].

The incidence of sexual harassment in the workplace among nurses has increased. The impact of sexual harassment also causes psychological problems such as fear, anxiety, and discomfort in the work environment. In addition, these problems have an impact on the work motivation of nurses and the desire of nurses to leave their jobs. This is in line with previous studies that showed that a safe and comfortable environment can improve the performance of nurses in providing nursing care to patients. One of the factors that increases the safety of nurses in the workplace is avoiding sexual violence.

### Limitation

This study was limited to analyzing sexual harassment by patients. Meanwhile, information regarding experiences of sexual harassment by other workers was not obtained. This study has other limitations, namely the number of samples (40 mental health nurses). In addition, this study was also limited to one hospital location, namely the Mental Hospital of West Java Province.

## 5. Conclusions

Sexual harassment among mental health nurses in mental hospitals, particularly in West Java, Indonesia, is an area of concern to be addressed. The sexual harassment that is felt by mental health nurses is physical and verbal. Experiences of sexual harassment among nurses include being kissed, hugged from behind, held by the breasts, and invited to have sexual intercourse. Nurses feel shocked and afraid and experience anxiety due to sexual harassment by patients with mental disorders. The experience of sexual harassment against psychiatric nurses also causes nurses to want to leave their jobs. The nurses who experience sexual harassment are mostly female nurses. This can be caused by the existence of power relations between men and women. Therefore, the prevention of sexual harassment can be facilitated by compatibility between the genders of patients and nurses. The implication of this study is that there is a foundation for health workers in managing nurses in hospitals to improve the quality of nurse performance. This study also recommends that a director of a mental hospital, who has responsibility for safety considerations, should develop protection and insurance programs for nurses to address sexual harassment. Follow-up treatment for nurses experiencing sexual harassment is important. It is proposed that a psychological rehabilitation program should be developed for nurses suffering from trauma. Further research should analyze the relationship of sexual harassment experience with the working alliance among nurses.

## Figures and Tables

**Table 1:** Socio-demographic characteristics of the nurses at the Mental Hospital of West Java Province (n = 40).

Socio-Demographic Characteristic		Frequency (n)	Percentage (%)
**Gender**			
	Male	10	25
	Female	30	75
**Education Level**			
	D III (Associate)	22	55
	S1 (Bachelor)	14	35
	S2 (Master and Specialist)	4	10
**Duration of Work**			
	1–10 years	11	27.5
	11–15 years	16	40
	16–20 years	8	20
	more 20 years	5	12.5
**The Longest Workplace**			
	Polyclinic/inpatient department	4	10
	Acute Room	11	27.5
	Chronic Room	14	35
	Emergency Room	6	15
	Drug Addiction and Rehabilitation Unit	5	12.5

## Data Availability

Not applicable.
